# Impact of Exposure to Benzodiazepines on Adverse Effects and Efficacy of PD‐1/PD‐L1 Blockade in Patients With Non‐Small Cell Lung Cancer

**DOI:** 10.1111/1759-7714.70081

**Published:** 2025-05-14

**Authors:** Kiyoshi Takagaki, Yoshiya Ohno, Taiichiro Otsuki, Aki Kubota, Takashi Kijima, Toshiyuki Tanaka

**Affiliations:** ^1^ Laboratory of Immunobiology School of Pharmacy, Hyogo Medical University Kobe Japan; ^2^ Department of Pharmacy Hyogo Medical University Hospital Nishinomiya Japan; ^3^ Department of Respiratory Medicine and Hematology School of Medicine, Hyogo Medical University Nishinomiya Japan; ^4^ Department of Thoracic Oncology School of Medicine, Hyogo Medical University Nishinomiya Japan; ^5^ Department of Biomedical Statistics and Bioinformatics Kyoto University Graduate School of Medicine Kyoto Japan

**Keywords:** benzodiazepines, concomitant baseline medication, immune checkpoint inhibitors, immune‐related adverse events, non‐small cell lung cancer

## Abstract

**Background:**

The impact of concomitant medications on immune‐related adverse events (irAEs) and immune checkpoint inhibitor (ICI) efficacy in non‐small cell lung cancer (NSCLC) remains unclear. Benzodiazepine receptor agonists (BZRAs), commonly prescribed for anxiety and insomnia in cancer care, may influence antitumor immunity via γ‐aminobutyric acid (GABA) signaling. Here, we retrospectively analyzed medical records of NSCLC patients treated with ICIs.

**Methods:**

In an initial exploratory analysis, BZRA use was significantly associated with a lower incidence of irAEs, prompting further evaluation. Propensity score matching (PSM) was performed to adjust for potential confounding factors. In the matched cohort, we assessed associations between BZRA use, irAE incidence, and ICI efficacy, as measured by progression‐free survival (PFS) and overall survival (OS).

**Results:**

In the matched cohort, BZRA use was significantly associated with a lower incidence of irAEs (OR 0.33, 95% CI: 0.13–0.80, *p* = 0.015). BZRA use was also linked to shorter PFS (HR 1.80, 95% CI: 1.13–2.86, *p* = 0.013), but not OS (HR 1.63, 95% CI: 0.95–2.81, *p* = 0.077). In subgroup analysis, among patients who developed irAEs, BZRA use was associated with shorter PFS (HR 2.69, 95% CI: 1.32–5.48, *p* = 0.007) and OS (HR 3.35, 95% CI: 1.40–8.04, *p* = 0.007), whereas no significant associations were observed in non‐irAE patients.

**Conclusion:**

BZRA use was associated with reduced irAE incidence and poorer ICI outcomes among patients who developed irAEs, suggesting potential immunosuppressive effects that may impair ICI efficacy in NSCLC.

## Graphical abstract

The use of benzodiazepine receptor agonists (BZRAs) was linked to a reduced incidence of irAEs in patients with NSCLC receiving anti‐PD‐1/PD‐L1 monotherapy. However, among those who developed irAEs, BZRA use was associated with shorter PFS and OS. These findings suggest that while BZRAs may lower the incidence of irAEs, they could also diminish the effectiveness of ICI therapy in NSCLC patients with irAEs.

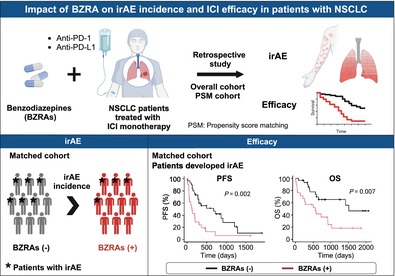

**Abbreviations**
AEC: absolute eosinophil count
BZRAs: benzodiazepine receptor agonists
CI: confidence interval
ECOG: eastern cooperative oncology group
EGFR: epidermal growth factor receptor
HR: hazard ratio
ICIs: immune checkpoint inhibitors
ILD: interstitial lung disease
irAEs: immune‐related adverse events
LMR: lymphocyte‐to‐monocyte ratio
NLR: neutrophil‐to‐lymphocyte ratio
NSCLC: non‐small cell lung cancer
OR: odds ratio
OS: overall survival
PD‐1: programmed cell death 1
PD‐L1: programmed death‐ligand 1
PFS: progression‐free survival
PPIs: proton pump inhibitors
PS: performance status
PSM: propensity score matching
SMD: standardized mean difference

## 1 Introduction

Immune checkpoint inhibitors (ICIs), including programmed cell death 1 (PD‐1) and programmed death‐ligand 1 (PD‐L1) inhibitors, have been used to treat various cancers, including non‐small cell lung cancer (NSCLC). As the primary drug therapy for patients with NSCLC without specific driver mutations or translocations, ICIs have shown potential to improve survival rates [1]. However, their efficacy remains limited, and their use is often constrained by immune‐related adverse events (irAEs), which complicate patient management and treatment continuity [2, 3].

As ICI therapy involves complex interactions within the immune system, there has been growing interest in understanding how concomitant medications influence immune modulation and treatment outcomes. Several retrospective studies have evaluated the impact of baseline medications, including corticosteroids, antibiotics, proton pump inhibitors (PPIs), and psychotropic drugs, on ICI therapeutic outcomes and safety profiles [4, 5, 6, 7, 8, 9, 10, 11, 12, 13]. However, the effects of these drugs vary depending on drug classes and patient populations, making it challenging to assess their overall impact on ICI therapy outcomes [9, 10, 11, 12, 13]. These variations make it difficult to determine the precise influence of specific concomitant drugs on both irAEs and treatment efficacy, highlighting the necessity further investigation.

Based on these considerations, we first conducted a retrospective analysis using patient medical records to examine the association between baseline medication use and the incidence of irAEs in patients with NSCLC undergoing ICI therapy. This analysis identified benzodiazepine receptor agonists (BZRAs) as significantly associated with a lower incidence of irAEs. Given the widespread use of BZRAs to manage anxiety and insomnia in cancer patients [14, 15] and our findings that BZRAs were associated with a lower incidence of irAEs, we further investigated whether BZRA use influences ICI treatment efficacy. Since γ‐aminobutyric acid (GABA) signaling, which is modulated through BZRA binding to GABA receptors, has been implicated in immune regulation [16], we hypothesized that BZRA use could impact clinical outcomes in patients with NSCLC receiving ICI therapy.

Here, we explored this hypothesis by retrospectively analyzing the impact of BZRA use on both irAE incidence and ICI efficacy, using data from the total cohort and a propensity score–matched cohort.

## 2 Methods

### 2.1 Ethical Approvement

This study followed the Declaration of Helsinki and was approved by the Ethics Committee of Hyogo Medical University (date of approval: May 19, 2021, approval number: 4161). The Ethics Committee waived the requirement for informed consent for retrospective analysis due to the study's non‐interventional design.

### 2.2 Patient Data Collection

**FIGURE 1:**
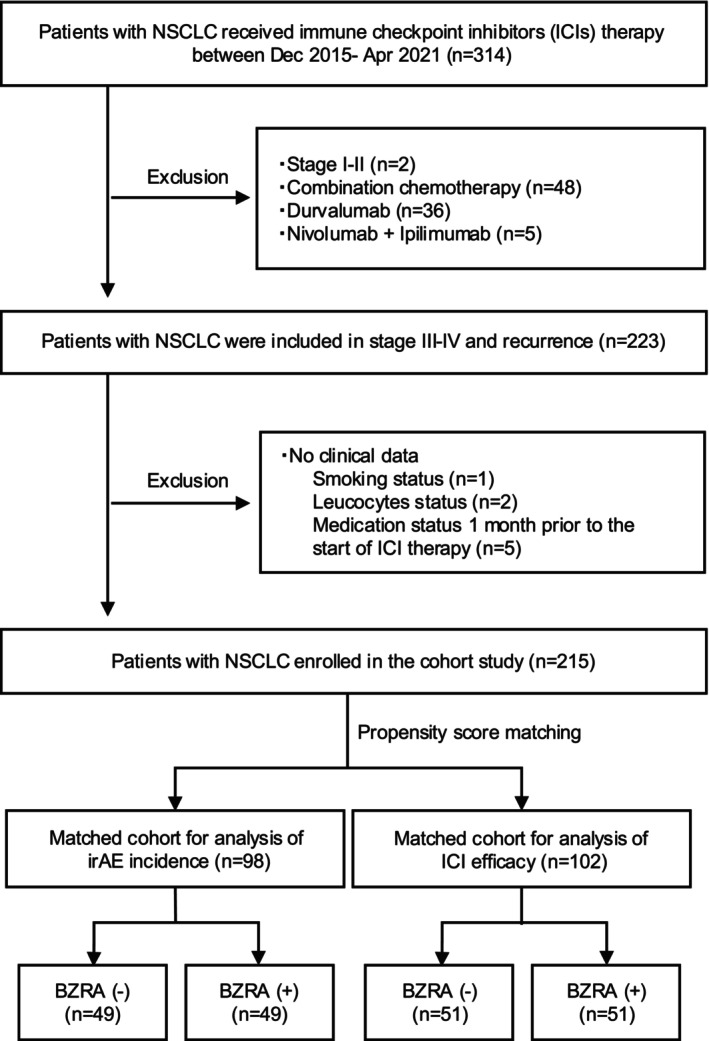
Flow chart of the study design. BZRA, benzodiazepine receptor agonist; NSCLC, non‐small cell lung cancer; ICIs, immune checkpoint inhibitors; irAE, immune‐related adverse event.

We retrospectively collected data from the medical records of patients with NSCLC who received ICI therapy at the Department of Respiratory Medicine, Hyogo Medical University Hospital, between December 1, 2015, and April 30, 2021. This study included patients who received nivolumab, pembrolizumab, or atezolizumab as ICI monotherapy for stage III‐IV/recurrent NSCLC. Patients were excluded if they had stage I–II disease; received chemotherapy, durvalumab (typically used as maintenance therapy following chemoradiotherapy), or combination therapy with nivolumab plus ipilimumab; or had incomplete clinical data (Figure 1). The following clinical data were obtained: age, sex, Eastern Cooperative Oncology Group (ECOG) performance status (PS), smoking status, histology, epidermal growth factor receptor (EGFR) mutation, PD‐L1 expression, ICI therapy, ICI treatment line, brain and liver metastasis, peripheral blood test results, and irAEs. The absolute eosinophil count (AEC) [17], neutrophil‐to‐lymphocyte ratio (NLR) [18], and lymphocyte‐to‐monocyte ratio (LMR) [19] were evaluated within 1 week of ICI therapy initiation. IrAEs were defined as skin disorders, adrenal insufficiency, colitis/enteritis/diarrhea, hepatopathy, hypopituitarism, interstitial lung disease (ILD)/pneumonia, rheumatoid arthritis/arthritis, thyroiditis/hypothyroidism, type I diabetes, and other adverse events diagnosed as irAEs by attending physicians and specialists. IrAEs were evaluated using the Common Terminology Criteria for Adverse Events v5.0.

### 2.3 Concomitant Baseline Medications

The following concomitant baseline medications were collected: angiotensin‐converting enzyme inhibitors/angiotensin II receptor blockers, beta‐blockers, antibiotics, corticosteroids, and BZRAs, which include both traditional benzodiazepines and Z‐drugs, antidepressants, dipeptidyl peptidase 4 inhibitors, metformin, statins, fibrates, histamine H1‐receptor blockers, H2 blockers, PPIs, nonsteroidal anti‐inflammatory drugs, opioids, probiotics, and immunosuppressants (Table S1). According to previous studies, concomitant baseline medications were defined as those used within 1 month before or after ICI treatment initiation [4, 5, 9, 11]. The use of medications as needed or those with topical effects was excluded. Medications prescribed in relation to irAE within 1 month of ICI administration were excluded from the concomitant baseline medication. Corticosteroid doses were corrected in prednisone equivalents; doses < 10 mg were considered within the physiological dose range based on previous studies [6, 7, 8, 9, 20, 21]. Patients in the corticosteroid < 10 mg group included those taking less than 10 mg of prednisone equivalent as well as those taking no corticosteroids.

### 2.4 Statistical Analysis

Univariable analysis was performed to compare factors, including concomitant baseline medications, between the irAE and non‐irAE groups using the Fisher exact and *χ* ^2^ tests. Additionally, the impact of concomitant baseline medications on the incidence of irAEs was evaluated using multivariable logistic regression analysis. Multivariable logistic regression analysis included variables with values of *p <* 0.1 in univariable analysis, clinically meaningful variables, and variables reported in previous studies.

Progression‐free survival (PFS) was defined as the time from ICI initiation to disease progression or death from any cause, and overall survival (OS) was defined as the time from ICI initiation to death from any cause. The cutoff for PFS and OS observations was November 1, 2021. The log‐rank test was used to compare Kaplan–Meier curves and evaluate the impact of irAE occurrence or concomitant baseline medications on PFS and OS. Multivariable Cox proportional hazards regression analysis was used to estimate the effect of patient characteristics and concomitant baseline medications on PFS and OS. Adjustment factors for the multivariable Cox proportional hazards regression analysis were comprehensively determined in consultation with the clinician, referencing previous studies, and considering clinically meaningful factors.

Propensity score matching (PSM) was performed using nearest neighbor matching with a 1:1 ratio, without replacement, and a caliper of 0.2. The standardized mean difference (SMD) was calculated before and after matching to assess pre‐matching imbalance and post‐matching balance. An SMD threshold of 0.15 was set, with factors showing an SMD < 0.15 considered to have low imbalance, and factors with an SMD ≥ 0.15 considered to have high imbalance. High‐imbalance factors were further adjusted in the multivariable logistic regression analysis for irAE incidence and in the Cox proportional hazards regression analysis for ICI efficacy in the matched cohort, respectively [22]. All statistical analyses were performed using EZR ver 1.60 [23]. The threshold for statistical significance was set at a two‐sided *p <* 0.05.

## 3 Results

### 3.1 Clinical Characteristics of the Study Cohort

Among the initially identified 314 patients with NSCLC who were treated with either ICI monotherapy or combination therapy, 215 patients were included. Table 1 displays detailed patient clinical characteristics and concomitant baseline medication use. The median age of the patients was 70 years (range: 30–92 years), with the majority (87%, *n* = 187) receiving anti‐PD‐1 therapy (nivolumab or pembrolizumab) and the remainder (13%, *n* = 28) receiving anti‐PD‐L1 therapy with atezolizumab. Notably, 54.9% (*n* = 118) of patients exhibited positive PD‐L1 expression, with 33.5% (*n* = 72) exhibiting high expression levels (≥ 50%). Biomarker analysis showed that 51.6% (*n* = 111) of patients had an AEC ≥ 175/μL, indicating a higher risk of irAEs, whereas 80.9% (*n* = 174) had an LMR > 1.6 and 28.8% (*n* = 62) had an NLR ≥ 5, both associated with a lower risk.

**TABLE 1:** Patient characteristics and concomitant baseline medications.

Characteristics		No.	(%)
Total		215	
Age	Median (range)	70.00	(30–92)
Sex	Male	162	(75.3)
	Female	53	(24.7)
ECOG PS	0–1	178	(82.8)
	≥ 2	37	(17.2)
Smoking status	Never	35	(16.3)
	Former/current	180	(83.7)
Histology	Non‐squamous cell carcinoma	141	(65.6)
	Squamous cell carcinoma	74	(34.4)
EGFR mutation	No	138	(64.2)
	Yes	16	(7.4)
	Unknown	61	(28.4)
PD‐L1 TPS	< 1%	46	(21.4)
	1%–49%	46	(21.4)
	≥ 50%	72	(33.5)
	Unknown	51	(23.7)
ICI therapy	Anti‐PD‐1	187	(87.0)
	Anti‐PD‐L1	28	(13.0)
ICI treatment line	1st	57	(26.5)
	≥ 2nd	158	(73.5)
Metastasis	Brain metastasis	33	(15.3)
	Liver metastasis	23	(10.7)
AEC	< 175/μL	104	(48.4)
	≥ 175/μL	111	(51.6)
LMR	≤ 1.6	41	(19.1)
	> 1.6	174	(80.9)
NLR	< 5	153	(71.2)
	≥ 5	62	(28.8)
Concomitant baseline medications			
ACEIs/ARBs		61	(28.4)
β blockers		18	(8.4)
Antibiotics		44	(20.5)
Corticosteroids^a^	< 10 mg	196	(91.2)
	≥ 10 mg	19	(8.8)
BZRAs		56	(26.0)
Antidepressants		11	(5.1)
DPP‐4 inhibitors		32	(14.9)
Metformin		7	(3.3)
Statins		53	(24.7)
Fibrates		3	(1.4)
H1 blockers		20	(9.3)
H2 blockers		15	(7.0)
PPIs		112	(52.1)
NSAIDs		89	(41.4)
Opioids		66	(30.7)
Probiotics		23	(10.7)
Immunosuppressants		3	(1.4)

Abbreviations: ACEIs, angiotensin‐converting enzyme inhibitors; AEC, absolute eosinophil count; ARBs, angiotensin receptor blockers; BZRAs, benzodiazepine receptor agonists; DPP‐4, dipeptidyl peptidase‐4; ECOG PS, Eastern Cooperative Oncology Group Performance Status; EGFR, epidermal growth factor receptor; ICI, immune checkpoint inhibitor; LMR, lymphocyte‐to‐monocyte ratio; NSAIDs, non‐steroidal anti‐inflammatory drugs; NLR, neutrophil‐to‐lymphocyte ratio; PD‐1, programmed cell death 1; PD‐L1, programmed death‐ligand 1; PPIs, proton pump inhibitors; TPS, tumor proportion score.

^a^ Prednisone equivalents.

The median follow‐up times for PFS and OS were 132 days (95% confidence interval [CI], 100–197) and 501 days (95% CI, 394–593), respectively.

### 3.2 Impact of Patient Characteristics and Concomitant Medications on the Incidence of irAEs

The incidence of irAEs is summarized in Table S2. Among 215 patients, 118 (54.9%) experienced at least one irAE of any grade. The most commonly reported all‐grade irAE was skin toxicity, which manifested as rash, eczema, acneiform rash, and urticaria, affecting 59 patients (27.4%). Severe irAEs, classified as grades 3–4, were observed in 30 patients, with ILD/pneumonia being the most prevalent, recorded in 14 patients (6.5%). No patient developed Grade 5 irAE. Patients experiencing irAEs demonstrated prolonged PFS (median 73 days vs. 280 days, *p <* 0.001) and OS (median 232 days vs. 721 days, *p <* 0.001) than those who did not experience irAEs [24, 25] (Figure S1).

Detailed statistical analyses were conducted to explore factors influencing the incidence of irAEs (Table 2). Univariable analysis showed that ECOG PS ≥ 2, anti‐PD‐L1 therapy, and NLR ≥ 5 were linked to a lower incidence of irAEs, whereas squamous cell carcinoma and AEC ≥ 175 were associated with a higher incidence. LMR had no significant effect. Additionally, the use of corticosteroids (≥ 10 mg), BZRAs, antidepressants, and opioids tends to decrease the likelihood of developing irAEs.

**TABLE 2:** Univariable and multivariable analyses of factors associated with irAEs.

Factor		Univariable analysis			Multivariable analysis	
		irAEs (%)	Non‐irAEs (%)	*p*	OR (95% CI)	*p*
Age	< 75	77 (35.8)	64 (29.8)	1		
	≥ 75	41 (19.1)	33 (15.3)			
Sex	Male	91 (42.3)	71 (33.0)	0.613		
	Female	27 (12.6)	26 (12.1)			
ECOG PS	0–1	105 (48.8)	73 (34.0)	0.014	Reference	
	≥ 2	13 (6.0)	24 (11.2)		0.45 (0.20–1.01)	0.054
Smoking status	Never	18 (8.4)	17 (7.9)	0.792		
	Former/current	100 (46.5)	80 (37.2)			
Histology	Non‐sq	70 (32.6)	71 (33.0)	0.047	Reference	
	Sq	48 (22.3)	26 (12.1)		1.83 (0.93–3.63)	0.081
EGFR mutation	No	71 (33.0)	67 (31.2)	0.118		
	Yes	7 (3.3)	9 (4.2)			
	Unknown	40 (18.6)	21 (9.8)			
PD‐L1 TPS	< 1%	23 (10.7)	23 (10.7)	0.429		
	1%–49%	22 (10.2)	24 (11.2)			
	≥ 50%	41 (19.1)	31 (14.4)			
	Unknown	32 (14.9)	19 (8.8)			
ICI therapy	Anti‐PD‐1	108 (50.2)	79 (36.7)	0.048	Reference	
	Anti‐PD‐L1	10 (4.7)	18 (8.4)		0.42 (0.17–1.05)	0.062
ICI treatment line	1st	37 (17.2)	20 (9.3)	0.105		
	≥ 2nd	81 (37.7)	77 (35.8)			
Brain metastasis	No	104 (48.4)	78 (36.3)	0.170		
	Yes	14 (6.5)	19 (8.8)			
Liver metastasis	No	108 (50.2)	84 (39.1)	0.346		
	Yes	10 (4.7)	13 (6.0)			
AEC	< 175/μL	47 (21.9)	57 (26.5)	0.009	Reference	
	≥ 175/μL	71 (33.0)	40 (18.6)		1.90 (1.05–3.42)	0.034
LMR	≤ 1.6	20 (9.3)	21 (9.8)	0.485		
	> 1.6	98 (45.6)	76 (35.3)			
NLR	< 5	92 (42.8)	61 (28.4)	0.023	Reference	
	≥ 5	26 (12.1)	36 (16.7)		0.41 (0.19–0.86)	0.019
Concomitant baseline medications						
ACEIs/ARBs	No	84 (39.1)	70 (32.6)	0.995		
	Yes	34 (15.8)	27 (12.6)			
β blockers	No	110 (51.2)	87 (40.5)	0.495		
	Yes	8 (3.7)	10 (4.7)			
Antibiotics	No	95 (44.2)	76 (35.3)	0.826		
	Yes	23 (10.7)	21 (9.8)			
Corticosteroids^a^	< 10 mg	112 (52.1)	84 (39.1)	0.058	Reference	
	≥ 10 mg	6 (2.8)	13 (6.0)		0.66 (0.21–2.11)	0.49
BZRAs	No	94 (43.7)	65 (30.2)	0.052	Reference	
	Yes	24 (11.2)	32 (14.9)		0.50 (0.25–0.98)	0.043
Antidepressants	No	115 (53.5)	89 (41.4)	0.069^b^	Reference	
	Yes	3 (1.4)	8 (3.7)		0.74 (0.15–3.64)	0.71
DPP‐4 inhibitors	No	97 (45.1)	86 (40.0)	0.258		
	Yes	21 (9.8)	11 (5.1)			
Metformin	No	112 (52.1)	96 (44.7)	0.131^b^		
	Yes	6 (2.8)	1 (0.5)			
Statins	No	86 (40.0)	76 (35.3)	0.443		
	Yes	32 (14.9)	21 (9.8)			
Fibrates	No	116 (54.0)	96 (44.7)	1^b^		
	Yes	2 (0.9)	1 (0.5)			
H1 blockers	No	106 (49.3)	89 (41.4)	0.805		
	Yes	12 (5.6)	8 (3.7)			
H2 blockers	No	113 (52.6)	87 (40.5)	0.142		
	Yes	5 (2.3)	10 (4.7)			
PPIs	No	59 (27.4)	44 (20.5)	0.589		
	Yes	59 (27.4)	53 (24.7)			
NSAIDs	No	70 (32.6)	56 (26.0)	0.923		
	Yes	48 (22.3)	41 (19.1)			
Opioids	No	88 (40.9)	61 (28.4)	0.089	Reference	
	Yes	30 (14.0)	36 (16.7)		0.87 (0.43–1.76)	0.70
Probiotics	No	105 (48.8)	87 (40.5)	1		
	Yes	13 (6.0)	10 (4.7)			
ISs	No	117 (54.4)	95 (44.2)	0.590^b^		
	Yes	1 (0.5)	2 (0.9)			

Abbreviations: ACEIs, angiotensin‐converting enzyme inhibitors; AEC, absolute eosinophil count; ARBs, angiotensin receptor blockers; BZRAs, benzodiazepine receptor agonists; CI, confidence interval; DPP‐4, dipeptidyl peptidase‐4; ECOG PS, Eastern Cooperative Oncology Group Performance Status; EGFR, epidermal growth factor receptor; ICI, immune checkpoint inhibitor; irAEs, immune‐related adverse events; ISs, immunosuppressants; LMR, lymphocyte‐to‐monocyte ratio; NSAIDs, non‐steroidal anti‐inflammatory drugs; NLR, neutrophil‐to‐lymphocyte ratio; OR, odds ratio; PD‐1, programmed cell death 1; PD‐L1, programmed death‐ligand 1; PPIs, proton pump inhibitors; Sq, Squamous cell carcinoma; TPS, tumor proportion score.

^a^ Prednisone equivalents.

^b^ Fisher's exact test.

A multivariable logistic regression analysis identified AEC ≥ 175 as a risk factor for irAEs (OR 1.90, 95% CI: 1.05–3.42, *p* = 0.034), while NLR ≥ 5 was linked to a lower incidence (OR 0.41, 95% CI: 0.19–0.86, *p* = 0.019). BZRA use was also associated with a lower incidence of irAEs (OR 0.50, 95% CI: 0.25–0.98, *p* = 0.043). In contrast, the use of corticosteroids, antidepressants, and opioids had no significant impact on the incidence of irAEs. In our subsequent analysis, we focused on BZRAs and excluded other concomitant baseline medications. Corticosteroids were also included to compare with BZRAs.

To mitigate potential biases in comparing irAE incidence rates between BZRA‐exposed and non‐exposed groups, we performed PSM using relevant patient characteristics (Age, Sex, ECOG PS, Smoking status, Histology, EGFR mutation, PD‐L1 TPS, ICI therapy, ICI treatment line, AEC, LMR, NLR, and Corticosteroid use) (Table 3). After matching, 49 pairs (*n* = 98) were obtained from the BZRA and non‐BZRA groups. The incidence of irAEs was observed in 21 out of 49 patients in the BZRA group and 31 out of 49 patients in the non‐BZRA group. After matching, a multivariable logistic regression analysis was performed, including PD‐L1 expression, ICI therapy, and NLR (SMD ≥ 0.15) as covariates. The analysis showed that BZRA use was significantly associated with a lower incidence of irAEs (OR 0.33, 95% CI: 0.13–0.80, *p* = 0.015), consistent with findings from the unmatched cohort (Table 4).

**TABLE 3:** Patient characteristics by BZRA use status before and after PSM to assess its impact on irAE incidence.

Factor		Before PSM				After PSM			
		BZRAs		*p*	SMD	BZRAs		*p*	SMD
		(−)	(+)			(−)	(+)		
		*n* = 159 (%)	*n* = 56 (%)			*n* = 49 (%)	*n* = 49 (%)		
Age	< 75	101 (63.5)	40 (71.4)	0.364	0.169	35 (71.4)	33 (67.3)	0.827	0.089
	≥ 75	58 (36.5)	16 (28.6)			14 (28.6)	16 (32.7)		
Sex	Male	119 (74.8)	43 (76.8)	0.913	0.045	37 (75.5)	37 (75.5)	1	< 0.001
	Female	40 (25.2)	13 (23.2)			12 (24.5)	12 (24.5)		
ECOG PS	0–1	130 (81.8)	48 (85.7)	0.64	0.107	42 (85.7)	42 (85.7)	1	< 0.001
	≥ 2	29 (18.2)	8 (14.3)			7 (14.3)	7 (14.3)		
Smoking status	Never	23 (14.5)	12 (21.4)	0.316	0.182	10 (20.4)	9 (18.4)	1	0.052
	Former/current	136 (85.5)	44 (78.6)			39 (79.6)	40 (81.6)		
Histology	Non‐sq	102 (64.2)	39 (69.6)	0.562	0.117	33 (67.3)	33 (67.3)	1	< 0.001
	Sq	57 (35.8)	17 (30.4)			16 (32.7)	16 (32.7)		
EGFR mutation	No	107 (67.3)	31 (55.4)	0.060^a^	0.343	30 (61.2)	31 (63.3)	1^a^	0.072
	Yes	8 (5.0)	8 (14.3)			5 (10.2)	4 (8.2)		
	Unknown	44 (27.7)	17 (30.4)			14 (28.6)	14 (28.6)		
PD‐L1 TPS	< 1%	32 (20.1)	14 (25.0)	0.702	0.185	9 (18.4)	13 (26.5)	0.584	0.284
	1%–49%	35 (22.0)	11 (19.6)			15 (30.6)	10 (20.4)		
	≥ 50%	56 (35.2)	16 (28.6)			15 (30.6)	14 (28.6)		
	Unknown	36 (22.6)	15 (26.8)			10 (20.4)	12 (24.5)		
ICI therapy	Anti‐PD‐1	138 (86.8)	49 (87.5)	1	0.021	45 (91.8)	42 (85.7)	0.524	0.195
	Anti‐PD‐L1	21 (13.2)	7 (12.5)			4 (8.2)	7 (14.3)		
ICI treatment line	1st	47 (29.6)	10 (17.9)	0.126	0.278	11 (22.4)	10 (20.4)	1	0.05
	≥ 2nd	112 (70.4)	46 (82.1)			38 (77.6)	39 (79.6)		
Brain metastasis	No	135 (84.9)	47 (83.9)	1	0.027	44 (89.8)	42 (85.7)	0.759	0.125
	Yes	24 (15.1)	9 (16.1)			5 (10.2)	7 (14.3)		
Liver metastasis	No	141 (88.7)	51 (91.1)	0.805	0.079	44 (89.8)	45 (91.8)	1^a^	0.071
	Yes	18 (11.3)	5 (8.9)			5 (10.2)	4 (8.2)		
AEC	< 175/μL	73 (45.9)	31 (55.4)	0.289	0.19	22 (44.9)	24 (49.0)	0.84	0.082
	≥ 175/μL	86 (54.1)	25 (44.6)			27 (55.1)	25 (51.0)		
LMR	≤ 1.6	30 (18.9)	11 (19.6)	1	0.02	11 (22.4)	9 (18.4)	0.802	0.101
	> 1.6	129 (81.1)	45 (80.4)			38 (77.6)	40 (81.6)		
NLR	< 5	111 (69.8)	42 (75.0)	0.572	0.116	32 (65.3)	36 (73.5)	0.511	0.178
	≥ 5	48 (30.2)	14 (25.0)			17 (34.7)	13 (26.5)		
Corticosteroids^b^	< 10 mg	146 (91.8)	50 (89.3)	0.588^a^	0.087	45 (91.8)	44 (89.8)	1^b^	0.071
	≥ 10 mg	13 (8.2)	6 (10.7)			4 (8.2)	5 (10.2)		

Abbreviations: AEC, absolute eosinophil count; BZRAs, benzodiazepine receptor agonists; ECOG PS, Eastern Cooperative Oncology Group Performance Status; EGFR, epidermal growth factor receptor; ICI, immune checkpoint inhibitor; irAE, immune‐related adverse event; LMR, lymphocyte‐to‐monocyte ratio; NLR, neutrophil‐to‐lymphocyte ratio; PD‐1, programmed cell death 1; PD‐L1, programmed death‐ligand 1; PSM, propensity score matching; SMD, standardized mean difference; Sq, squamous cell carcinoma; TPS, tumor proportion score.

^a^ Fisher exact test.

^b^ Prednisone equivalents.

**TABLE 4:** Multivariable analysis of the impact of BZRA use on the incidence of irAEs in the matched cohort.

Factor		Matched cohort (*n* = 98)	
		OR (95% CI)	*p*
PD‐L1 TPS	1%–49%	0.46 (0.13–1.64)	0.23
	≥ 50%	0.92 (0.26–3.23)	0.90
	Unknown	0.89 (0.24–3.32)	0.86
ICI therapy	Anti‐PD‐L1	0.69 (0.17–2.77)	0.60
NLR	≥ 5	0.22 (0.08–0.61)	0.004
BZRAs	Yes	0.33 (0.13–0.80)	0.015

Abbreviations: BZRAs, benzodiazepine receptor agonists; CI, confidence interval; ICI, immune checkpoint inhibitor; irAEs, immune‐related adverse events; NLR, neutrophil‐to‐lymphocyte ratio; OR, odds ratio; PD‐L1, programmed death‐ligand 1; TPS, tumor proportion score.

### 3.3 Impact of Patient Characteristics and the Concomitant Use of BZRAs on the Efficacy of ICI Therapy in Patients With NSCLC

#### 3.3.1 The Overall Impact on the Efficacy of ICIs

**FIGURE 2:**
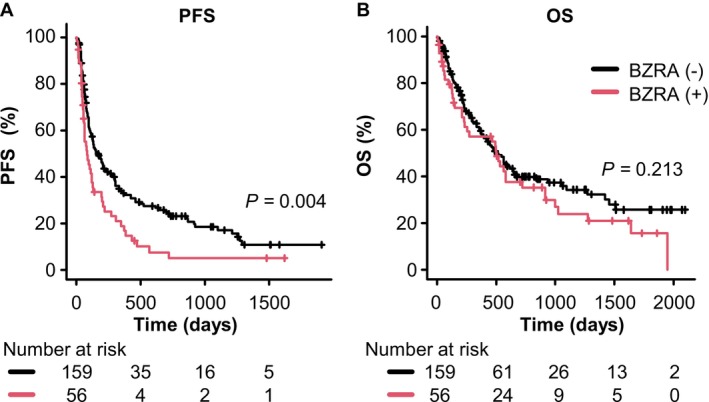
Kaplan–Meier curves for the effects of BZRA use on (A) PFS and (B) OS in patients with NSCLC treated with ICIs. BZRA, benzodiazepine receptor agonist; NSCLC, non‐small cell lung cancer; ICIs, immune checkpoint inhibitors; OS, overall survival; PFS, progression‐free survival.

To explore the impact of BZRA use on the clinical outcomes of ICI therapy, we used the Kaplan–Meier method to analyze PFS and OS. This analysis included all 215 patients, and it was observed that patients using BZRAs experienced significantly shorter PFS than those who did not (median PFS: 84 days vs. 161 days, *p =* 0.004). However, the difference in OS between the two groups was not significant, with median OS of 501 days in the BZRAs group and 494 days in the non‐BZRA group (*p =* 0.213) (Figure 2A,B).

A multivariable Cox proportional hazards regression analysis was conducted to evaluate the impact of patient characteristics and concomitant use of BZRAs on the efficacy of ICIs (Table 5). This analysis involved 215 patients, with the factors adjusted, as outlined in Table S3. Patients with ECOG PS ≥ 2 had shorter PFS and OS (Table 5). Similarly, NLR ≥ 5 was associated with shorter PFS (HR 1.63, 95% CI: 1.12–2.38, *p* = 0.012) and OS (HR 1.74, 95% CI: 1.12–2.70, *p* = 0.014). Patients exhibiting positive PD‐L1 expression, particularly those with high expression levels (PD‐L1 ≥ 50%), showed markedly improved survival rates with significantly longer PFS (HR 0.17, 95% CI: 0.11–0.29, *p <* 0.001) and OS (HR 0.56, 95% CI: 0.34–0.94, *p =* 0.027). The use of BZRAs did not significantly affect OS (HR 1.13, 95% CI: 0.75–1.71, *p =* 0.56), although it was notably associated with shorter PFS (HR 1.63, 95% CI: 1.13–2.35, *p =* 0.009).

**TABLE 5:** Multivariable analysis of the impact of BZRA use on PFS and OS.

Factor^a^		NSCLC (*n* = 215)			
		PFS		OS	
		HR (95% CI)	*p*	HR (95% CI)	*p*
Age	≥ 75	0.83 (0.58–1.20)	0.32	1.21 (0.80–1.82)	0.37
Sex	Female	0.99 (0.68–1.43)	0.94	0.72 (0.46–1.13)	0.15
ECOG PS	≥ 2	2.35 (1.48–3.75)	< 0.001	2.44 (1.48–4.02)	< 0.001
Histology	Sq	0.88 (0.60–1.29)	0.51	0.95 (0.62–1.46)	0.82
PD‐L1 TPS	1%–49%	0.37 (0.23–0.60)	< 0.001	0.96 (0.56–1.64)	0.87
	≥ 50%	0.17 (0.11–0.29)	< 0.001	0.56 (0.34–0.94)	0.027
	Unknown	0.32 (0.20–0.50)	< 0.001	0.74 (0.43–1.26)	0.26
Brain metastasis	Yes	0.92 (0.59–1.46)	0.74	0.87 (0.51–1.48)	0.61
Liver metastasis	Yes	1.22 (0.72–2.07)	0.45	1.55 (0.89–2.71)	0.12
irAEs	Yes	0.44 (0.31–0.62)	< 0.001	0.56 (0.38–0.84)	0.005
AEC	≥ 175/μL	0.77 (0.55–1.06)	0.11	0.76 (0.53–1.10)	0.15
NLR	≥ 5	1.63 (1.11–2.38)	0.012	1.74 (1.12–2.70)	0.014
Corticosteroids^b^	≥ 10 mg	1.92 (1.07–3.45)	0.029	1.12 (0.56–2.25)	0.75
BZRAs	Yes	1.63 (1.13–2.35)	0.009	1.13 (0.75–1.71)	0.56

Abbreviations: AEC, absolute eosinophil count; BZRAs, benzodiazepine receptor agonists; CI, confidence interval; ECOG PS, Eastern Cooperative Oncology Group Performance Status; HR, hazard ratio; irAEs, immune‐related adverse events; NLR, neutrophil‐to‐lymphocyte ratio; NSCLC, non‐small cell lung cancer; OS, overall survival; PD‐L1, programmed death‐ligand 1; PFS, progression‐free survival; Sq, squamous cell carcinoma; TPS, tumor proportion score.

^a^ Multivariable Cox proportional hazards regression analysis adjusted for variables shown in Table S3 that were associated with the incidence of irAEs and clinically meaningful variables determined in consultation with clinicians.

^b^ Prednisone equivalents.

### 3.4 Subgroup Analysis Based on irAE Status

**FIGURE 3:**
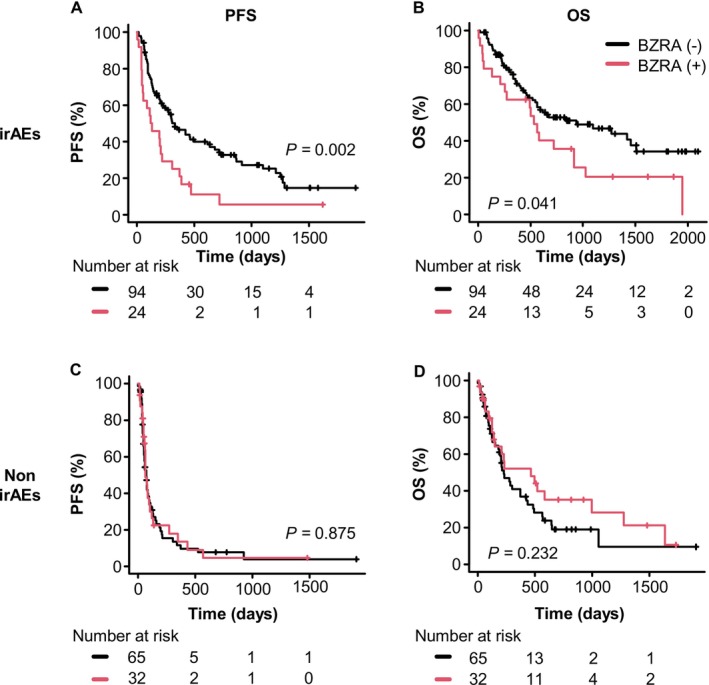
Kaplan–Meier curves for the effects of BZRA use on PFS and OS in patients with NSCLC treated with ICIs stratified by irAE status. Effects of BZRA use on (A) PFS and (B) OS in the irAE group, and (C) PFS and (D) OS in the non‐irAE group. BZRA, benzodiazepine receptor agonist; ICIs, immune checkpoint inhibitors; irAEs, immune‐related adverse events; NSCLC, non‐small cell lung cancer; OS, overall survival; PFS, progression‐free survival.

To examine the impact of BZRA use on clinical outcomes of ICI therapy based on irAE status, we compared Kaplan–Meier curves of PFS and OS by separating patients into irAE and non‐irAE groups (Figure 3). Among patients who developed irAEs, those using BZRAs showed significantly shorter PFS (median: 126.5 days vs. 332 days, *p =* 0.002) and OS (median: 533 days vs. 938 days, *p =* 0.041) compared to those not using BZRAs (Figure 3A,B). Conversely, in patients without irAEs, BZRA use was not significantly associated with PFS (median: 67 days vs. 73 days, *p =* 0.875) or OS (median: 466 days vs. 231 days, *p =* 0.232) (Figure 3C,D).

Multivariable Cox proportional hazards regression analysis was performed for subgroups based on irAE status (Table 6). This analysis was adjusted for the factors outlined in Table S3. Table 6 shows the stratified analysis by separating the patients into groups based on the absence or presence of irAEs. BZRA use was significantly associated with shorter PFS (HR 2.33, 95% CI: 1.36–3.94, *p =* 0.002) and OS (HR 2.18, 95% CI: 1.21–3.96, *p =* 0.01) in patients with irAEs, compared to those without BZRA use. In contrast, BZRA use did not significantly affect PFS and OS in patients without irAEs. In the group of patients with irAEs, ECOG PS ≥ 2 and NLR ≥ 5 were associated with shorter PFS and OS, whereas PD‐L1 expression was associated with longer PFS. In the group of patients without irAEs, ECOG PS ≥ 2 and NLR ≥ 5 were associated with shorter PFS, and high PD‐L1 expression (≥ 50%) was associated with longer PFS (HR 1.08, 95% CI: 0.63–1.83, *p =* 0.79) and OS (HR 0.67, 95% CI: 0.37–1.21, *p =* 0.19).

**TABLE 6:** Multivariable analysis of the impact of BZRA use on PFS and OS stratified by irAEs.

Factor^a^		irAEs (*n* = 118)				Non‐irAEs (*n* = 97)			
		PFS		OS		PFS		OS	
		HR (95% CI)	*p*	HR (95% CI)	*p*	HR (95% CI)	*p*	HR (95% CI)	*p*
Age	≥ 75	1.26 (0.75–2.10)	0.38	1.35 (0.74–2.47)	0.33	0.60 (0.34–1.06)	0.077	1.21 (0.66–2.20)	0.54
Sex	Female	1.06 (0.62–1.81)	0.83	1.04 (0.55–1.98)	0.91	0.70 (0.39–1.26)	0.24	0.53 (0.28–1.03)	0.063
ECOG PS	≥ 2	2.69 (1.25–5.81)	0.011	5.02 (2.17–11.62)	< 0.001	2.13 (1.13–4.04)	0.02	1.93 (0.97–3.82)	0.06
Histology	Sq	0.67 (0.39–1.13)	0.13	0.67 (0.37–1.23)	0.19	1.19 (0.65–2.15)	0.57	1.57 (0.82–2.98)	0.17
PD‐L1 TPS	1%–49%	0.42 (0.21–0.86)	0.018	1.32 (0.56–3.13)	0.52	0.26 (0.13–0.52)	< 0.001	0.66 (0.31–1.41)	0.28
	≥ 50%	0.19 (0.10–0.39)	< 0.001	0.86 (0.39–1.89)	0.70	0.19 (0.09–0.39)	< 0.001	0.41 (0.20–0.87)	0.021
	Unknow	0.31 (0.16–0.58)	< 0.001	0.70 (0.32–1.51)	0.36	0.25 (0.12–0.51)	< 0.001	0.80 (0.37–1.76)	0.58
Brain metastasis	Yes	1.17 (0.57–2.40)	0.66	0.82 (0.31–2.18)	0.70	1.19 (0.60–2.36)	0.62	0.96 (0.46–1.99)	0.92
Liver metastasis	Yes	1.34 (0.61–2.97)	0.47	2.21 (0.97–5.04)	0.059	1.59 (0.71–3.56)	0.26	1.31 (0.54–3.14)	0.55
AEC	≥ 175/μL	0.82 (0.52–1.31)	0.41	0.91 (0.53–1.57)	0.74	0.66 (0.37–1.15)	0.14	0.67 (0.38–1.17)	0.16
NLR	≥ 5	1.82 (1.01–3.25)	0.044	2.24 (1.17–4.28)	0.015	1.86 (1.09–3.19)	0.023	1.36 (0.72–2.59)	0.35
Corticosteroids^b^	≥ 10 mg	0.50 (0.14–1.82)	0.29	0.68 (0.17–2.71)	0.58	6.01 (2.66–13.57)	< 0.001	2.08 (0.79–5.44)	0.14
BZRAs	Yes	2.33 (1.38–3.94)	0.002	2.18 (1.21–3.96)	0.01	1.08 (0.63–1.83)	0.79	0.67 (0.37–1.21)	0.19

Abbreviations: AEC, absolute eosinophil count; BZRAs, benzodiazepine receptor agonists; CI, confidence interval; ECOG PS, Eastern Cooperative Oncology Group Performance Status; HR, hazard ratio; irAEs, immune‐related adverse events; NLR, neutrophil‐to‐lymphocyte ratio; OS, overall survival; PD‐L1, programmed death‐ligand 1; PFS, progression‐free survival; Sq, squamous cell carcinoma; TPS, tumor proportion score.

^a^ Multivariable Cox proportional hazards regression analysis adjusted for variables shown in Table S3 that were associated with the incidence of irAEs and clinically meaningful variables determined in consultation with clinicians.

^b^ Prednisone equivalents.

### 3.5 Matched Cohort Analysis

To address potential confounding from BZRA exposure, we conducted PSM based on relevant patient characteristics: age, sex, ECOG PS, smoking status, histology, EGFR mutation, PD‐L1 expression, ICI therapy, ICI treatment line, brain and liver metastasis, NLR, and corticosteroid use (Table 7). This matching yielded 51 pairs in the BZRA and non‐BZRA groups. Of these patients, 51 developed irAEs and 51 did not, with 21 irAE cases and 30 non‐irAE cases using BZRAs. After matching, some imbalances remained in smoking status (SMD: 0.207), PD‐L1 expression (SMD: 0.170), liver metastasis (SMD: 0.191) and LMR (SMD: 0.152).

**TABLE 7:** Patient characteristics by BZRA use status before and after PSM to assess its impact on ICI efficacy.

Factor		Before PSM				After PSM			
		BZRAs		*p*	SMD	BZRAs		*p*	SMD
		(−)	(+)			(−)	(+)		
		*n* = 159 (%)	*n* = 56 (%)			*n* = 51 (%)	*n* = 51 (%)		
Age	< 75	101 (63.5)	40 (71.4)	0.364	0.169	37 (72.5)	35 (68.6)	0.828	0.086
	≥ 75	58 (36.5)	16 (28.6)			14 (27.5)	16 (31.4)		
Sex	Male	119 (74.8)	43 (76.8)	0.913	0.045	41 (80.4)	38 (74.5)	0.636	0.141
	Female	40 (25.2)	13 (23.2)			10 (19.6)	3 (25.5)		
ECOG PS	0–1	130 (81.8)	48 (85.7)	0.64	0.107	44 (86.3)	43 (84.3)	1	0.055
	≥ 2	29 (18.2)	8 (14.3)			7 (13.7)	8 (15.7)		
Smoking status	Never	23 (14.5)	12 (21.4)	0.316	0.182	7 (13.7)	11 (21.6)	0.436	0.207
	Former/current	136 (85.5)	44 (78.6)			44 (86.3)	40 (78.4)		
Histology	Non‐sq	102 (64.2)	39 (69.6)	0.562	0.117	33 (64.7)	34 (66.7)	1	0.041
	Sq	57 (35.8)	17 (30.4)			18 (35.3)	17 (33.3)		
EGFR mutation	No	107 (67.3)	31 (55.4)	0.060^a^	0.343	31 (60.8)	31 (60.8)	1^a^	0.075
	Yes	8 (5.0)	8 (14.3)			5 (9.8)	4 (7.8)		
	Unknown	44 (27.7)	17 (30.4)			15 (29.4)	16 (31.4)		
PD‐L1 TPS	< 1%	32 (20.1)	14 (25.0)	0.702	0.185	13 (25.5)	12 (23.5)	0.866	0.170
	1%–49%	35 (22.0)	11 (19.6)			10 (19.6)	11 (21.6)		
	≥ 50%	56 (35.2)	16 (28.6)			13 (25.5)	16 (31.4)		
	Unknown	36 (22.6)	15 (26.8)			15 (29.4)	12 (23.5)		
ICI therapy	Anti‐PD‐1	138 (86.8)	49 (87.5)	1	0.021	47 (92.2)	45 (88.2)	0.739	0.132
	Anti‐PD‐L1	21 (13.2)	7 (12.5)			4 (7.8)	6 (11.8)		
ICI treatment line	1st	47 (29.6)	10 (17.9)	0.126	0.278	10 (19.6)	10 (19.6)	1	< 0.001
	≥ 2nd	112 (70.4)	46 (82.1)			41 (80.4)	41 (80.4)		
Brain metastasis	No	135 (84.9)	47 (83.9)	1	0.027	42 (82.4)	43 (84.3)	1	0.053
	Yes	24 (15.1)	9 (16.1)			9 (17.6)	8 (15.7)		
Liver metastasis	No	141 (88.7)	51 (91.1)	0.805	0.079	44 (86.3)	47 (92.2)	0.523	0.191
	Yes	18 (11.3)	5 (8.9)			7 (13.7)	4 (7.8)		
AEC	< 175/μL	73 (45.9)	31 (55.4)	0.289	0.19	25 (49.0)	26 (51.0)	1	0.039
	≥ 175/μL	86 (54.1)	25 (44.6)			26 (51.0)	25 (49.0)		
LMR	≤ 1.6	30 (18.9)	11 (19.6)	1	0.02	8 (15.7)	11 (21.6)	0.611	0.152
	> 1.6	129 (81.1)	45 (80.4)			43 (84.3)	40 (78.4)		
NLR	< 5	111 (69.8)	42 (75.0)	0.572	0.116	40 (78.4)	37 (72.5)	0.645	0.137
	≥ 5	48 (30.2)	14 (25.0)			11 (21.6)	14 (27.5)		
Corticosteroids^b^	< 10 mg	146 (91.8)	50 (89.3)	0.588^a^	0.087	45 (88.2)	45 (88.2)	1	< 0.001
	≥ 10 mg	13 (8.2)	6 (10.7)			6 (11.8)	6 (11.8)		

Abbreviations: AEC, absolute eosinophil count; BZRAs, benzodiazepine receptor agonists; ECOG PS, Eastern Cooperative Oncology Group Performance Status; EGFR, epidermal growth factor receptor; ICI, immune checkpoint inhibitor; LMR, lymphocyte‐to‐monocyte ratio; NLR, neutrophil‐to‐lymphocyte ratio; PD‐1, programmed cell death 1; PD‐L1, programmed death‐ligand 1; PSM, propensity score matching; SMD, standardized mean difference; Sq, squamous cell carcinoma; TPS, tumor proportion score.

^a^ Fisher exact test.

^b^ Prednisone equivalents.

**FIGURE 4:**
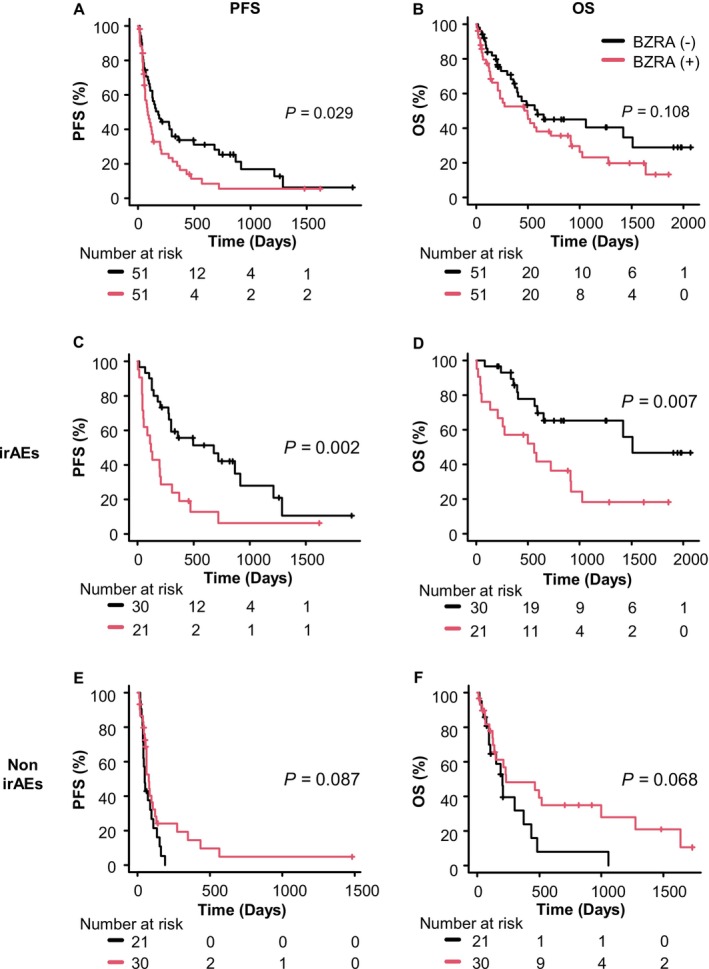
Kaplan–Meier curves for the effect of BZRA use on PFS and OS in patients with NSCLC treated with ICIs in the matched cohorts. Effects of BZRA use on (A) PFS and (B) OS in the entire matched cohort, (C) PFS and (D) OS in the irAE group, and (E) PFS and (F) OS in the non‐irAE group. BZRA, benzodiazepine receptor agonist; ICIs, immune checkpoint inhibitors; irAEs, immune‐related adverse events; NSCLC, non‐small cell lung cancer; OS, overall survival; PFS, progression‐free survival.

The Kaplan–Meier curves for the entire matched cohort (Figure 4A,B) illustrate a significantly shorter PFS for patients with BZRA use compared to those without BZRA use (median PFS: 90 days vs. 182 days, *p* = 0.029), while no significant difference was observed in OS (median OS: 497 days vs. 593 days, *p* = 0.108). In the subgroup analysis based on irAE status, among patients who developed irAEs, Kaplan–Meier curves (Figure 4C,D) show that patients with BZRA use experienced significantly shorter PFS and OS compared to those without BZRA (median PFS: 122 days vs. 681 days, *p* = 0.002; median OS: 562 days vs. 1510 days, *p* = 0.007). In contrast, BZRA use did not significantly impact PFS or OS among patients without irAEs (Figure 4E,F).

Multivariable Cox proportional hazards regression analysis in the matched cohort (*n* = 102), adjusting for residual imbalances (Table 8, upper section), showed that BZRA use was significantly associated with shorter PFS (HR 1.80, 95% CI: 1.13–2.86, *p* = 0.013) but not OS (HR 1.63, 95% CI: 0.95–2.81, *p* = 0.077). In the subgroup analysis based on irAE status (Table 8, lower section), among irAE patients, BZRA use was associated to both shorter PFS (HR 2.69, 95% CI: 1.32–5.48, *p* = 0.007) and OS (HR 3.35, 95% CI: 1.40–8.04, *p* = 0.007). BZRA use had no significant effect on PFS or OS in non‐irAE patients. These findings in the matched cohort were consistent with those observed in the unmatched cohort.

**TABLE 8:** Multivariable analysis of the impact of BZRA use on the efficacy of ICI in the matched cohort.

Factor		Matched cohort (*n* = 102)			
		PFS		OS	
		HR (95% CI)	*p*	HR (95% CI)	*p*
Matched cohort (*n* = 102)					
(BZRAs: No *n* = 51/Yes *n* = 51)					
Smoking status	Former/current	0.90 (0.49–1.64)	0.72	1.73 (0.79–3.80)	0.17
PD‐L1 TPS	1%–49%	0.51 (0.27–0.97)	0.04	1.42 (0.67–3.01)	0.35
	≥ 50%	0.21 (0.10–0.42)	< 0.001	0.78 (0.38–1.61)	0.51
	Unknown	0.33 (0.18–0.62)	< 0.001	0.87 (0.43–1.74)	0.69
Liver metastasis	Yes	1.09 (0.48–2.44)	0.84	1.80 (0.79–4.13)	0.16
LMR	> 1.6	0.39 (0.22–0.69)	0.001	0.34 (0.18–0.64)	< 0.001
BZARs	Yes	1.80 (1.13–2.86)	0.013	1.63 (0.95–2.81)	0.077
*Subgroup analysis based on irAE status*					
irAE group (*n* = 51)					
(BZRAs: No *n* = 30/Yes *n* = 21)					
Smoking status	Former/current	0.83 (0.31–2.19)	0.71	1.03 (0.31–3.37)	0.97
PD‐L1 TPS	1%–49%	0.48 (0.17–1.36)	0.17	1.72 (0.48–6.23)	0.41
	≥ 50%	0.13 (0.04–0.38)	< 0.001	1.19 (0.39–3.62)	0.76
	Unknown	0.15 (0.05–0.43)	< 0.001	0.56 (0.16–1.89)	0.35
Liver metastasis	Yes	1.01 (0.30–3.48)	0.98	2.15 (0.58–7.98)	0.25
LMR	> 1.6	0.25 (0.08–0.82)	0.022	0.13 (0.04–0.46)	0.002
BZARs	Yes	2.69 (1.32–5.48)	0.007	3.35 (1.40–8.04)	0.007
Non‐irAE group (*n* = 51)					
(BZRAs: No *n* = 21/Yes *n* = 30)					
Smoking status	Former/current	1.08 (0.46–2.53)	0.86	2.06 (0.62–6.84)	0.24
PD‐L1 TPS	1%–49%	0.54 (0.21–1.41)	0.21	1.67 (0.61–4.59)	0.32
	≥ 50%	0.30 (0.09–1.05)	0.059	0.72 (0.21–2.45)	0.60
	Unknown	1.01 (0.43–2.40)	0.98	2.63 (0.99–7.00)	0.053
Liver metastasis	Yes	2.03 (0.62–6.61)	0.24	2.19 (0.67–7.13)	0.19
LMR	> 1.6	0.68 (0.32–1.47)	0.33	0.60 (0.26–1.38)	0.23
BZARs	Yes	0.87 (0.41–1.82)	0.71	0.64 (0.29–1.41)	0.27

Abbreviations: BZRAs, benzodiazepine receptor agonists; CI, confidence interval; HR, hazard ratio; irAE, immune‐related adverse event; LMR, lymphocyte‐to‐monocyte ratio; OS, overall survival; PD‐L1, programmed death‐ligand 1; PFS, progression‐free survival; TPS, tumor proportion score.

## 4 Discussion

In this study, we analyzed the effects of concomitant baseline medication use, including BZRAs, on the incidence of irAEs and clinical outcomes of ICI monotherapy in patients with NSCLC. In our initial analysis using the total cohort, BZRA use was associated with a lower incidence of irAEs; this association was confirmed in a matched cohort constructed for irAE analysis. In a separate matched cohort for survival analysis, BZRA use was associated with shorter PFS and OS among patients who developed irAEs, whereas no such association was observed in those without irAEs.

Previous studies have indicated that psychotropic drugs may adversely affect ICI's therapeutic efficacy [4, 11]. Our study highlighted the effects of BZRAs, demonstrating that they inhibited PFS and OS in patients with NSCLC with irAEs. BZRAs, commonly prescribed for insomnia and anxiety, are known for their potential risks, including delirium, cognitive dysfunction, and dependency, especially with prolonged use [14, 15]. Considering these risks and our results, BZRAs should be used with caution in patients with NSCLC receiving ICI treatment, especially in those who have developed irAEs. Alternatives, such as orexin receptor antagonists and melatonin receptor agonists, may be preferable for managing sleep disorders and anxiety in these patients. However, further studies are required to understand their impact and efficacy in this context fully.

In addition, this study explored a broader spectrum of medication interactions. Although antibiotics and PPIs, along with corticosteroids, have been reported to affect ICI efficacy variably [5, 6, 7, 11, 12, 13], our data did not show a significant impact of these drugs. This discrepancy might be attributed to the characteristics of our patient cohort: a large proportion (73.5%) received ICI therapy as second‐line treatment and only a minority (33.5%) exhibited high PD‐L1 expression (≥ 50%) [12, 13]. Previous studies have reported that using more than 10 mg of prednisone equivalent for palliative treatment at the start of ICI therapy was associated with poor treatment outcomes [20, 21]. The significant association between corticosteroid use and shorter PFS observed in this cohort, particularly in patients without irAEs, may be attributed to the subgroup of patients requiring palliative care. These patients typically had a poorer prognosis, and the early termination of ICI treatment before the onset of irAEs could have influenced their outcomes.

The emerging interest in the relationship between the microbiome and ICI efficacy suggests that dysbiosis induced by certain medications can influence treatment outcomes [26]. Several psychotropic drugs, including those structurally similar to BZRAs, have been reported to affect the intestinal microbiome [27], potentially altering the efficacy of ICI therapy. In addition to this indirect effect, BZRAs may directly influence ICI outcomes through GABA‐A receptor activation [28], which plays a critical role not only in the central nervous system but also in immune cells [16]. GABA signaling may modulate immune responses by inhibiting T cell proliferation [29] and alleviating inflammation [30, 31]. Furthermore, a study has shown that lorazepam, a specific type of BZRA, induces IL‐6 expression in cancer‐associated fibroblasts via G protein‐coupled receptor 68, which is associated with reduced survival of patients with pancreatic cancer [32]. These findings suggest that BZRAs, through both microbiome‐mediated and direct immune effects, may reduce the efficacy of ICIs.

This study had several limitations. First, as a single‐center retrospective study, the risk of selection bias limits the generalizability of our findings. Additionally, physician treatment choices and variability in follow‐up periods introduce potential confounding factors, which may lead to bias. Second, the relatively small sample size was not determined based on a priori power calculations, which may affect the reliability of statistical inferences. Furthermore, a formal statistical hypothesis was not established a priori to justify the sample size, which could limit the interpretability of our findings. Accordingly, the statistical power limitations should be considered when interpreting the findings, particularly for analyses with small effect sizes. Third, although multivariate analysis was performed to account for potential confounders, the number of covariates relative to the sample size may have led to overfitting and statistical instability. Fourth, the absence of an external validation cohort prevents us from assessing the reproducibility of our findings, necessitating cautious interpretation.

During the revision process, we identified a recent independent study by Montégut et al., which demonstrated that BZRAs may suppress the therapeutic efficacy of ICI [33]. This external evidence aligns with our findings and supports BZRA's influence on ICI treatment outcomes. However, the study by Montégut et al. did not investigate the impact of BZRAs on irAEs. In contrast, our study examined both efficacy and irAEs within a single dataset, offering additional insights into the potential effects of BZRAs in ICI‐treated patients with NSCLC. These findings highlight the need for larger‐scale, multi‐center prospective studies to validate these observations and further assess the therapeutic and safety implications of BZRA use in ICI therapy. Future studies should collect standardized and comprehensive data on patient characteristics, treatment duration, and biomarker expression levels, with particular emphasis on factors influencing the effects of BZRAs in ICI therapy. Expanding these analyses across diverse clinical settings will be essential to refine patient selection criteria and optimize treatment strategies for NSCLC patients receiving ICIs.

## 5 Conclusion

In patients with NSCLC, baseline BZRA use may be associated with a lower incidence of irAEs during ICI therapy. BZRAs might also reduce the efficacy of ICI therapy in these patients with irAEs. Reviewing BZRA use before initiating ICI therapy could improve therapeutic outcomes in this population.

## Author Contributions

**Kiyoshi Takagaki:** conceptualization, validation, formal analysis, investigation, data curation, writing. **Yoshiya Ohno:** conceptualization, validation, writing, funding acquisition. **Taiichiro Otsuki:** resources, validation. **Aki Kubota:** methodology, formal analysis. **Takashi Kijima:** resources, supervision, funding acquisition. **Toshiyuki Tanaka:** conceptualization, writing, visualization, supervision, project administration, funding acquisition. All authors discussed the results, reviewed the manuscript, and approved its submission.

## Conflicts of Interest

The authors declare the following financial interests/personal relationships that may be considered potential competing interests: Takashi Kijima reports receiving honoraria for lectures from Chugai Pharmaceutical, Merck Sharp & Dohme, and Ono Pharmaceutical outside of the submitted work. The other authors have no conflicts of interest to declare.

## Supporting information


**Figure S1.** Kaplan–Meier curves for (A) PFS and (B) OS in patients with NSCLC treated with ICIs stratified by the presence or absence of irAEs. ICIs, immune checkpoint inhibitors; irAEs, immune‐related adverse events; NSCLC, non‐small cell lung cancer; OS, overall survival; PFS, progression‐free survival.

**Table S1.** List of concomitant baseline medications.

**Table S2.** Immune‐related adverse events (irAEs) according to category and grade.

**Table S3.** Summary of factors adjusted for multivariable Cox proportional hazards regression analysis.

## Data Availability

The data that support the findings of this study are available from the corresponding author upon reasonable request.

## References

[1] F. Zhou , M. Qiao , and C. Zhou , “The Cutting‐Edge Progress of Immune‐Checkpoint Blockade in Lung Cancer,” Cellular & Molecular Immunology 18, no. 2 (2021): 279–293, 10.1038/s41423-020-00577-5. 33177696 PMC8027847

[2] M. A. Postow , R. Sidlow , and M. D. Hellmann , “Immune‐Related Adverse Events Associated With Immune Checkpoint Blockade,” New England Journal of Medicine 378, no. 2 (2018): 158–168, 10.1056/NEJMra1703481. 29320654

[3] G. Morad , B. A. Helmink , P. Sharma , and J. A. Wargo , “Hallmarks of Response, Resistance, and Toxicity to Immune Checkpoint Blockade,” Cell 184, no. 21 (2021): 5309–5337, 10.1016/j.cell.2021.09.020. 34624224 PMC8767569

[4] E. Pérez‐Ruiz , J. Jiménez‐Castro , M. A. Berciano‐Guerrero , et al., “Impact of Intestinal Dysbiosis‐Related Drugs on the Efficacy of Immune Checkpoint Inhibitors in Clinical Practice,” Clinical & Translational Oncology 22, no. 10 (2020): 1778–1785, 10.1007/s12094-020-02315-9. 32096143

[5] M. Chalabi , A. Cardona , D. R. Nagarkar , et al., “Efficacy of Chemotherapy and Atezolizumab in Patients With Non‐Small‐Cell Lung Cancer Receiving Antibiotics and Proton Pump Inhibitors: Pooled Post Hoc Analyses of the OAK and POPLAR Trials,” Annals of Oncology 31, no. 4 (2020): 525–531, 10.1016/j.annonc.2020.01.006. 32115349

[6] A. Cortellini , M. di Maio , O. Nigro , et al., “Differential Influence of Antibiotic Therapy and Other Medications on Oncological Outcomes of Patients With Non‐Small Cell Lung Cancer Treated With First‐Line Pembrolizumab Versus Cytotoxic Chemotherapy,” Journal for Immunotherapy of Cancer 9, no. 4 (2021): e002421, 10.1136/jitc-2021-002421. 33827906 PMC8031700

[7] S. Buti , M. Bersanelli , F. Perrone , et al., “Effect of Concomitant Medications With Immune‐Modulatory Properties on the Outcomes of Patients With Advanced Cancer Treated With Immune Checkpoint Inhibitors: Development and Validation of a Novel Prognostic Index,” European Journal of Cancer 142 (2021): 18–28, 10.1016/j.ejca.2020.09.033. 33212418

[8] K. C. Arbour , L. Mezquita , N. Long , et al., “Impact of Baseline Steroids on Efficacy of Programmed Cell Death‐1 and Programmed Death‐Ligand 1 Blockade in Patients With Non‐Small‐Cell Lung Cancer,” Journal of Clinical Oncology 36, no. 28 (2018): 2872–2878, 10.1200/JCO.2018.79.0006. 30125216

[9] M. Svaton , M. Zemanova , P. Zemanova , et al., “Impact of Concomitant Medication Administered at the Time of Initiation of Nivolumab Therapy on Outcome in Non‐Small Cell Lung Cancer,” Anticancer Research 40, no. 4 (2020): 2209–2217, 10.21873/anticanres.14182. 32234916

[10] K. Miura , Y. Sano , S. Niho , et al., “Impact of Concomitant Medication on Clinical Outcomes in Patients With Advanced Non‐Small Cell Lung Cancer Treated With Immune Checkpoint Inhibitors: A Retrospective Study,” Thoracic Cancer 12, no. 13 (2021): 1983–1994, 10.1111/1759-7714.14001. 33990133 PMC8258365

[11] M. Kostine , E. Mauric , A. Tison , et al., “Baseline Co‐Medications May Alter the Anti‐Tumoural Effect of Checkpoint Inhibitors as Well as the Risk of Immune‐Related Adverse Events,” European Journal of Cancer 157 (2021): 474–484, 10.1016/j.ejca.2021.08.036. 34649118

[12] H. Kawachi , T. Yamada , M. Tamiya , et al., “Concomitant Proton Pump Inhibitor Use With Pembrolizumab Monotherapy vs Immune Checkpoint Inhibitor Plus Chemotherapy in Patients With Non‐Small Cell Lung Cancer,” JAMA Network Open 6, no. 7 (2023): e2322915, 10.1001/jamanetworkopen.2023.22915. 37432682 PMC10336622

[13] N. Ochi , E. Ichihara , N. Takigawa , et al., “The Effects of Antibiotics on the Efficacy of Immune Checkpoint Inhibitors in Patients With Non‐Small‐Cell Lung Cancer Differ Based on PD‐L1 Expression,” European Journal of Cancer 149 (2021): 73–81, 10.1016/j.ejca.2021.02.040. 33838391

[14] D. S. Baldwin , K. Aitchison , A. Bateson , et al., “Benzodiazepines: Risks and Benefits. A Reconsideration,” Journal of Psychopharmacology 27, no. 11 (2013): 967–971, 10.1177/0269881113503509. 24067791

[15] M. E. Hirschtritt , M. Olfson , and K. Kroenke , “Balancing the Risks and Benefits of Benzodiazepines,” Journal of the American Medical Association 325, no. 4 (2021): 347–348, 10.1001/jama.2020.22106. 33416846

[16] A. K. Bhandage and A. Barragan , “GABAergic Signaling by Cells of the Immune System: More the Rule Than the Exception,” Cellular and Molecular Life Sciences 78, no. 15 (2021): 5667–5679, 10.1007/s00018-021-03881-z. 34152447 PMC8316187

[17] R. Bai , N. Chen , X. Chen , et al., “Analysis of Characteristics and Predictive Factors of Immune Checkpoint Inhibitor‐Related Adverse Events,” Cancer Biology & Medicine 18, no. 4 (2021): 1118–1133, 10.20892/j.issn.2095-3941.2021.0052. 34259422 PMC8610160

[18] L. Peng , Y. Wang , F. Liu , et al., “Peripheral Blood Markers Predictive of Outcome and Immune‐Related Adverse Events in Advanced Non‐Small Cell Lung Cancer Treated With PD‐1 Inhibitors,” Cancer Immunology, Immunotherapy 69, no. 9 (2020): 1813–1822, 10.1007/s00262-020-02585-w. 32350592 PMC7413896

[19] S. Egami , H. Kawazoe , H. Hashimoto , et al., “Peripheral Blood Biomarkers Predict Immune‐Related Adverse Events in Non‐Small Cell Lung Cancer Patients Treated With Pembrolizumab: A Multicenter Retrospective Study,” Journal of Cancer 12, no. 7 (2021): 2105–2112, 10.7150/jca.53242. 33754009 PMC7974524

[20] B. Ricciuti , S. E. Dahlberg , A. Adeni , L. M. Sholl , M. Nishino , and M. M. Awad , “Immune Checkpoint Inhibitor Outcomes for Patients With Non‐Small‐Cell Lung Cancer Receiving Baseline Corticosteroids for Palliative Versus Nonpalliative Indications,” Journal of Clinical Oncology 37, no. 22 (2019): 1927–1934, 10.1200/JCO.19.00189. 31206316

[21] M. Skribek , K. Rounis , S. Afshar , et al., “Effect of Corticosteroids on the Outcome of Patients With Advanced Non‐Small Cell Lung Cancer Treated With Immune‐Checkpoint Inhibitors,” European Journal of Cancer 145 (2021): 245–254, 10.1016/j.ejca.2020.12.012. 33419647

[22] T. L. Nguyen , G. S. Collins , J. Spence , et al., “Double‐Adjustment in Propensity Score Matching Analysis: Choosing a Threshold for Considering Residual Imbalance,” BMC Medical Research Methodology 17, no. 1 (2017): 78, 10.1186/s12874-017-0338-0. 28454568 PMC5408373

[23] Y. Kanda , “Investigation of the Freely Available Easy‐To‐Use Software “EZR” for Medical Statistics,” Bone Marrow Transplantation 48, no. 3 (2013): 452–458, 10.1038/bmt.2012.244. 23208313 PMC3590441

[24] K. Haratani , H. Hayashi , Y. Chiba , et al., “Association of Immune‐Related Adverse Events With Nivolumab Efficacy in Non‐Small‐Cell Lung Cancer,” JAMA Oncology 4, no. 3 (2018): 374–378, 10.1001/jamaoncol.2017.2925. 28975219 PMC6583041

[25] B. Shankar , J. Zhang , A. R. Naqash , et al., “Multisystem Immune‐Related Adverse Events Associated With Immune Checkpoint Inhibitors for Treatment of Non‐Small Cell Lung Cancer,” JAMA Oncology 6, no. 12 (2020): 1952–1956, 10.1001/jamaoncol.2020.5012. 33119034 PMC7596677

[26] R. K. Weersma , A. Zhernakova , and J. Fu , “Interaction Between Drugs and the Gut Microbiome,” Gut 69, no. 8 (2020): 1510–1519, 10.1136/gutjnl-2019-320204. 32409589 PMC7398478

[27] L. Maier , M. Pruteanu , M. Kuhn , et al., “Extensive Impact of Non‐Antibiotic Drugs on Human Gut Bacteria,” Nature 555, no. 7698 (2018): 623–628, 10.1038/nature25979. 29555994 PMC6108420

[28] U. Rudolph and F. Knoflach , “Beyond Classical Benzodiazepines: Novel Therapeutic Potential of GABAA Receptor Subtypes,” Nature Reviews. Drug Discovery 10, no. 9 (2011): 685–697, 10.1038/nrd3502. 21799515 PMC3375401

[29] E. L. Sparrow , S. James , K. Hussain , S. A. Beers , M. S. Cragg , and Y. D. Bogdanov , “Activation of GABA(A) Receptors Inhibits T Cell Proliferation,” PLoS One 16, no. 5 (2021): e0251632, 10.1371/journal.pone.0251632. 34014994 PMC8136847

[30] B. Zhang , A. Vogelzang , M. Miyajima , et al., “B Cell‐Derived GABA Elicits IL‐10+ Macrophages to Limit Anti‐Tumour Immunity,” Nature 599, no. 7885 (2021): 471–476, 10.1038/s41586-021-04082-1. 34732892 PMC8599023

[31] D. Huang , Y. Wang , J. W. Thompson , et al., “Cancer‐Cell‐Derived GABA Promotes β‐Catenin‐Mediated Tumour Growth and Immunosuppression,” Nature Cell Biology 24, no. 2 (2022): 230–241, 10.1038/s41556-021-00820-9. 35145222 PMC8852304

[32] A. C. Cornwell , A. A. Tisdale , S. Venkat , et al., “Lorazepam Stimulates IL6 Production and is Associated With Poor Survival Outcomes in Pancreatic Cancer,” Clinical Cancer Research 29, no. 18 (2023): 3793–3812, 10.1158/1078-0432.CCR-23-0547. 37587561 PMC10502465

[33] L. Montégut , L. Derosa , M. Messaoudene , et al., “Benzodiazepines Compromise the Outcome of Cancer Immunotherapy,” Oncoimmunology 13, no. 1 (2024): 2413719, 10.1080/2162402x.2024.2413719. 39381589 PMC11459736

