# Correction to “Impact of Exposure to Benzodiazepines on Adverse Effects and Efficacy of PD‐1/PD‐L1 Blockade in Patients With Non‐Small Cell Lung Cancer”

**DOI:** 10.1111/1759-7714.70143

**Published:** 2025-08-07

K. Takagaki, Y. Ohno, T. Otsuki, A. Kubota, T. Kijima, T. Tanaka, “Impact of Exposure to Benzodiazepines on Adverse Effects and Efficacy of PD‐1/PD‐L1 Blockade in Patients With Non‐Small Cell Lung Cancer,” *Thoracic Cancer* 16, (2025): e70081. https://doi.org/10.1111/1759‐7714.70081.

On the 3.4 Subgroup Analysis Based on irAE Status heading, second paragraph, the below final sentence is incorrect.

In the group of patients without irAEs, ECOG PS ≥ 2 and NLR ≥ 5 were associated with shorter PFS, and high PD‐L1 expression (≥ 50%) was associated with longer PFS (HR 1.08, 95% CI: 0.63–1.83, *p* = 0.79) and OS (HR 0.67, 95% CI: 0.37–1.21, *p* = 0.19).

It should be corrected to as follows.

In the group of patients without irAEs, ECOG PS ≥ 2 and NLR ≥ 5 were associated with shorter PFS, and high PD‐L1 expression (≥ 50%) was associated with longer PFS (HR 0.19, 95% CI: 0.09–0.39, *p* < 0.001) and OS (HR 0.41, 95% CI: 0.20–0.87, *p* = 0.021).

In Table 7, the value for sex: female in the After PSM: BZRA (+) column was incorrect. Below is the correct Table 7.

**TABLE 7:** Patient characteristics by BZRA use status before and after PSM to assess its impact on ICI efficacy.

Factor		Before PSM				After PSM			
		BZRAs				BZRAs			
		(−)	(+)	*p*	SMD	(−)	(+)	*p*	SMD
		*n* = 159 (%)	*n* = 56 (%)			*n* = 51 (%)	*n* = 51 (%)		
Age	< 75	101 (63.5)	40 (71.4)	0.364	0.169	37 (72.5)	35 (68.6)	0.828	0.086
	≥ 75	58 (36.5)	16 (28.6)			14 (27.5)	16 (31.4)		
Sex	Male	119 (74.8)	43 (76.8)	0.913	0.045	41 (80.4)	38 (74.5)	0.636	0.141
	Female	40 (25.2)	13 (23.2)			10 (19.6)	13 (25.5)		
ECOG PS	0–1	130 (81.8)	48 (85.7)	0.64	0.107	44 (86.3)	43 (84.3)	1	0.055
	≥ 2	29 (18.2)	8 (14.3)			7 (13.7)	8 (15.7)		
Smoking status	Never	23 (14.5)	12 (21.4)	0.316	0.182	7 (13.7)	11 (21.6)	0.436	0.207
	Former/current	136 (85.5)	44 (78.6)			44 (86.3)	40 (78.4)		
Histology	Non‐sq	102 (64.2)	39 (69.6)	0.562	0.117	33 (64.7)	34 (66.7)	1	0.041
	Sq	57 (35.8)	17 (30.4)			18 (35.3)	17 (33.3)		
EGFR mutation	No	107 (67.3)	31 (55.4)	0.060^a^	0.343	31 (60.8)	31 (60.8)	1^a^	0.075
	Yes	8 (5.0)	8 (14.3)			5 (9.8)	4 (7.8)		
	Unknown	44 (27.7)	17 (30.4)			15 (29.4)	16 (31.4)		
PD‐L1 TPS	< 1%	32 (20.1)	14 (25.0)	0.702	0.185	13 (25.5)	12 (23.5)	0.866	0.170
	1%–49%	35 (22.0)	11 (19.6)			10 (19.6)	11 (21.6)		
	≥ 50%	56 (35.2)	16 (28.6)			13 (25.5)	16 (31.4)		
	Unknown	36 (22.6)	15 (26.8)			15 (29.4)	12 (23.5)		
ICI therapy	Anti‐PD‐1	138 (86.8)	49 (87.5)	1	0.021	47 (92.2)	45 (88.2)	0.739	0.132
	Anti‐PD‐L1	21 (13.2)	7 (12.5)			4 (7.8)	6 (11.8)		
ICI treatment line	1st	47 (29.6)	10 (17.9)	0.126	0.278	10 (19.6)	10 (19.6)	1	< 0.001
	≥ 2nd	112 (70.4)	46 (82.1)			41 (80.4)	41 (80.4)		
Brain metastasis	No	135 (84.9)	47 (83.9)	1	0.027	42 (82.4)	43 (84.3)	1	0.053
	Yes	24 (15.1)	9 (16.1)			9 (17.6)	8 (15.7)		
Liver metastasis	No	141 (88.7)	51 (91.1)	0.805	0.079	44 (86.3)	47 (92.2)	0.523	0.191
	Yes	18 (11.3)	5 (8.9)			7 (13.7)	4 (7.8)		
AEC	< 175/μL	73 (45.9)	31 (55.4)	0.289	0.19	25 (49.0)	26 (51.0)	1	0.039
	≥ 175/μL	86 (54.1)	25 (44.6)			26 (51.0)	25 (49.0)		
LMR	≤ 1.6	30 (18.9)	11 (19.6)	1	0.02	8 (15.7)	11 (21.6)	0.611	0.152
	> 1.6	129 (81.1)	45 (80.4)			43 (84.3)	40 (78.4)		
NLR	< 5	111 (69.8)	42 (75.0)	0.572	0.116	40 (78.4)	37 (72.5)	0.645	0.137
	≥ 5	48 (30.2)	14 (25.0)			11 (21.6)	14 (27.5)		
Corticosteroids^b^	< 10 mg	146 (91.8)	50 (89.3)	0.588^a^	0.087	45 (88.2)	45 (88.2)	1	< 0.001
	≥ 10 mg	13 (8.2)	6 (10.7)			6 (11.8)	6 (11.8)		

Abbreviations: AEC, absolute eosinophil count; BZRAs, benzodiazepine receptor agonists; ECOG PS, Eastern Cooperative Oncology Group Performance Status; EGFR, epidermal growth factor receptor; ICI, immune checkpoint inhibitor; LMR, lymphocyte‐to‐monocyte ratio; NLR, neutrophil‐to‐lymphocyte ratio; PD‐1, programmed cell death 1; PD‐L1, programmed death‐ligand 1; PSM, propensity score matching; SMD, standardized mean difference; Sq, squamous cell carcinoma; TPS, tumor proportion score.

^a^ Fisher exact test.

^b^ Prednisone equivalents.

We apologize for these errors.

