# Supplementary material for: Impact of Exposure to Benzodiazepines on Adverse Effects and Efficacy of PD‐1/PD‐L1 Blockade in Patients With Non‐Small Cell Lung Cancer
Source: Thorac Cancer. 2025 May 14;16(9):e70081. doi: 10.1111/1759-7714.70081 (PMC12077927; doi:10.1111/1759-7714.70081)
Supplement: Supplementary file 1 — Figure S1. Kaplan–Meier curves for (A) PFS and (B) OS in patients with NSCLC treated with ICIs stratified by the presence or absence of irAEs. ICIs, immune checkpoint inhibitors; irAEs, immune‐related adverse events; NSCLC, non‐small cell lung cancer; OS, overall survival; PFS, progression‐free survival. [file TCA-16-e70081-s004.pdf]

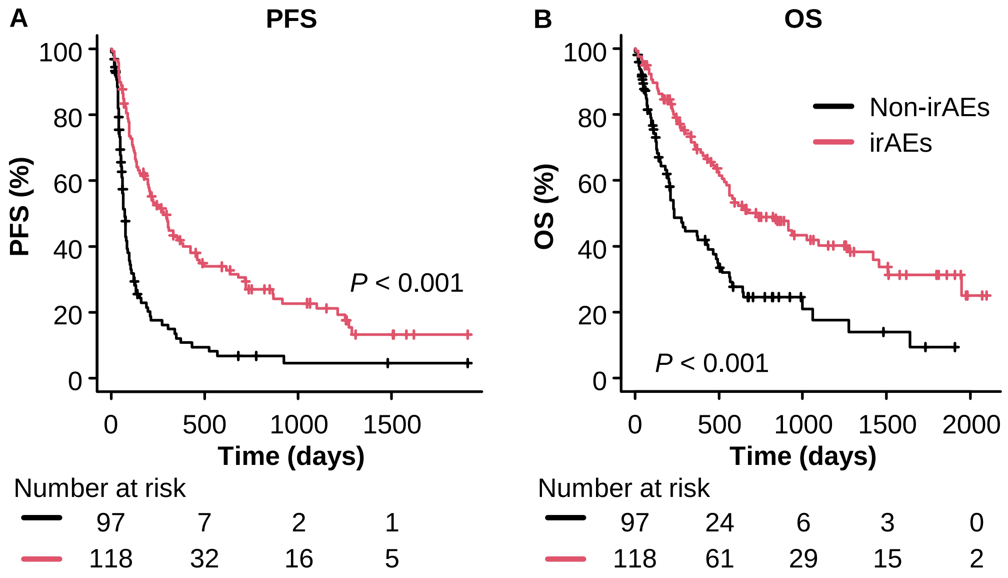

Supplementary Figure 1. Kaplan–Meier curves for (A) PFS and (B) OS in patients with NSCLC treated with ICIs stratified by the presence or absence of irAEs.

Abbreviations: NSCLC, non-small cell lung cancer; ICIs, immune checkpoint inhibitors; irAEs, immune-related adverse events; OS, overall survival; PFS, progression-free survival.
