# Supplementary material for: Impact of Exposure to Benzodiazepines on Adverse Effects and Efficacy of PD‐1/PD‐L1 Blockade in Patients With Non‐Small Cell Lung Cancer
Source: Thorac Cancer. 2025 May 14;16(9):e70081. doi: 10.1111/1759-7714.70081 (PMC12077927; doi:10.1111/1759-7714.70081)
Supplement: Supplementary file 2 — Table S1. List of concomitant baseline medications. [file TCA-16-e70081-s003.pdf]

Supplementary Table 1. List of concomitant baseline medications.

Concomitant baseline medications	Drug name†, ‡
ACEIs	Imidapril (3), Enalapril (2)
ARBs	Telmisartan (11), Valsartan (11), Candesartan (11), Irbesartan (9), Olmesartan (7), Azilsartan (5), Losartan (4)
β blockers	Bisoprolol (9), Carvedilol (8), Atenolol (1)
Antibiotics	Clarithromycin (6), Sulfamethoxazole/Trimethoprim (6), Tazobactam/Piperacillin (5), Azithromycin (4), Cefcapene (4), Garenoxacin (4), Lascufloxacin (4), Levofloxacin (4), Cefozopran (3), Meropenem (3), Minocycline (3), Sitafloxacin (3), Ampicillin/Sulbactam (2), Ampicillin (1), Amoxicillin/Clavulanate (1), Cefaclor (1), Cefazolin (1), Clindamycin (1), Moxifloxacin (1), Sultamicillin (1)
Antibiotics Series	Quinolones (14), β-lactams (10), Macrolides (8), Sulfamethoxazole/Trimethoprim (6), Carbapenems (3), Tetracyclines (3)
Corticosteroids	Prednisolone (18), Betamethasone (16), Dexamethasone (5), Hydrocortisone (2), Methylprednisolone (1)
BZRAs	Zolpidem (25), Etizolam (10), Brotizolam (8), Estazolam (4), Eszopiclone (4), Triazolam (4), Clonazepam (3), Flunitrazepam (3), Loflazepate (3), Lorazepam (2), Clotiazepam (1), Cloxazolam (1), Nitrazepam (1)
Antidepressants	Duloxetine (8), Mirtazapine (2), Fluvoxamine (1), Paroxetine (1), Sertraline (1)
DPP-4 inhibitors	Sitagliptin (14), Alogliptin (7), Linagliptin (7), Teneligliptin (4), Vildagliptin (4), Anagliptin (2), Saxagliptin (1)
Metformin	Metformin (7)
Statin	Rosuvastatin (25), Atorvastatin (14), Pitavastatin (7), Pravastatin (4), Fluvastatin (2), Simvastatin (1),
Fibrates	Bezafibrate (3)
H1 blockers	Fexofenadine (8), Olopatadine (6), Bepotastine (1), d-Chlorpheniramine (1), Desloratadine (1), Diphenhydramine (1), Ebastine (1), Levocetirizine (1), Loratadine (1)
H2 blockers	Famotidine (8), Lafutidine (4), Ranitidine (2), Nizatidine (1)
PPIs	Esomeprazole (72), Lansoprazole (20), Vonoprazan (17), Rabeprazole (4), Omeprazole (1)
NSAIDs	Loxoprofen (39), Naproxen (21), Aspirin (20), Celecoxib (13), Meloxicam (2), Diclofenac (1), Lornoxicam (1)
Opioids	Oxycodone (25), Tramadol (15), Codeine phosphate (14), Fentanyl (6), Hydromorphone (3), Morphine (2), Dihydrocodeine phosphate (3), Methadone (1), Tapentadol (1)
Probiotics	Clostridium butyricum MIYAIRI [MIYA-BM] (10), Lactomin/ butyric acid bacteria/ Amylolytic bacillus [Bio-three] (7), Lactomin amylolytic bacillus [Biofermin] (5), Bifidobacterium [LAC-B Granular Powder N] (1)
Immunosuppressants	Methotrexate (2), Tacrolimus (1)

Abbreviations: ACEIs, angiotensin-converting enzyme inhibitors; ARBs, angiotensin receptor blockers; BZRAs, benzodiazepine receptor agonists; DPP-4, dipeptidyl peptidase-4; NSAIDs, non-steroidal anti-inflammatory drugs; PPIs, proton pump inhibitors.

† Includes medications taken at the same time and before and after a drug change.

‡ Numbers represent real numbers.
