# Supplementary material for: Impact of Exposure to Benzodiazepines on Adverse Effects and Efficacy of PD‐1/PD‐L1 Blockade in Patients With Non‐Small Cell Lung Cancer
Source: Thorac Cancer. 2025 May 14;16(9):e70081. doi: 10.1111/1759-7714.70081 (PMC12077927; doi:10.1111/1759-7714.70081)
Supplement: Supplementary file 3 — Table S2. Immune‐related adverse events (irAEs) according to category and grade. [file TCA-16-e70081-s002.pdf]

Supplementary Table 2. Immune-related adverse events (irAEs) according to category and grade.

Category	Patients (%) † All grade	Grade1–2 ‡	Grade3–4
Any irAEs	118 (54.9)	104 (48.4)	30 (14.0)
Adrenal insufficiency	6 (2.8)	4 (1.9)	2 (0.9)
Colitis/ enteritis/ diarrhea	24 (11.2)	21 (9.8)	3 (1.4)
Hepatopathy	9 (4.2)	6 (2.8)	3 (1.4)
Hypopituitarism	7 (3.3)	4 (1.9)	3 (1.4)
ILD/ pneumonia	35 (16.3)	21 (9.8)	14 (6.5)
Rash/ Eczema/ Rash acneiform/ Urticaria	59 (27.4)	57 (26.5)	2 (0.9)
Rheumatoid arthritis/ arthritis	4 (1.9)	4 (1.9)	NA
Thyroiditis/ hypothyroidism	28 (13.0)	28 (13.0)	NA
Type 1 diabetes	1 (0.5)	NA	1 (0.5)
Others			
Interstitial nephritis	2 (0.9)	NA	2 (0.9)
Cholangitis	1 (0.5)	NA	1 (0.5)
Eosinophilia	1 (0.5)	1 (0.5)	NA
Myelodysplastic syndromes	1 (0.5)	1 (0.5)	NA
Myositis	1 (0.5)	1 (0.5)	NA
Peripheral neuropathy	1 (0.5)	1 (0.5)	NA
Renal dysfunction	1 (0.5)	1 (0.5)	NA

Abbreviations: irAEs, immune-related adverse events; ILD, interstitial lung disease; NA, not applicable

† Percentages may add up to more than 100 because some patients experienced more than 1 event.

‡ 16 patients had overlapping Grade 1–2 and Grade 3–4 irAEs.
