# Supplementary material for: Impact of Exposure to Benzodiazepines on Adverse Effects and Efficacy of PD‐1/PD‐L1 Blockade in Patients With Non‐Small Cell Lung Cancer
Source: Thorac Cancer. 2025 May 14;16(9):e70081. doi: 10.1111/1759-7714.70081 (PMC12077927; doi:10.1111/1759-7714.70081)
Supplement: Supplementary file 4 — Table S3. Summary of factors adjusted for multivariable Cox proportional hazards regression analysis. [file TCA-16-e70081-s001.pdf]

Supplementary Table 3. Summary of factors adjusted for multivariable Cox proportional hazards regression analysis.

Characteristics		irAEs		Non-irAEs	
Total		118		97	
Age (%)	<75	77	(65.3)	64	(66.0)
	≥75	41	(34.7)	33	(34.0)
Sex (%)	Male	91	(77.1)	71	(73.2)
	Female	27	(22.9)	26	(26.8)
ECOG PS (%)	0–1	105	(89.0)	73	(75.3)
	≥2	13	(11.0)	24	(24.7)
Histology (%)	Non-squamous cell carcinoma	70	(59.3)	71	(73.2)
	Squamous cell carcinoma	48	(40.7)	26	(26.8)
PD-L1 TPS (%)	<1%	23	(19.5)	23	(23.7)
	1–49%	22	(18.6)	24	(24.7)
	≥50%	41	(34.7)	31	(32.0)
	Unknown	32	(27.1)	19	(19.6)
Brain metastasis (%)	No	104	(88.1)	78	(80.4)
	Yes	14	(11.9)	19	(19.6)
Liver metastasis (%)	No	108	(91.5)	84	(86.6)
	Yes	10	(8.5)	13	(13.4)
AEC (%)	<175/ μL	47	(39.8)	57	(58.8)
	≥175/ μL	71	(60.2)	40	(41.2)
NLR (%)	<5	92	(78.0)	61	(62.9)
	≥5	26	(22.0)	36	(37.1)
BZRAs (%)	No	94	(79.7)	65	(67.0)
	Yes	24	(20.3)	32	(33.0)
Corticosteroids[†] (%)	<10 mg	112	(94.9)	84	(86.6)
	≥10 mg	6	(5.1)	13	(13.4)

Abbreviations: AEC, absolute eosinophil count; BZRAs, benzodiazepine receptor agonists; ECOG PS, Eastern Cooperative Oncology Group Performance Status; irAEs, immune-related adverse events; NLR, neutrophil-to-lymphocyte ratio; PD-L1, programmed death-1 ligand 1; TPS, tumor proportion score.

† Prednisone equivalent
